# Strategic integration of marketing and supply chain functions for superior customer experience: Insights from logistics startups under Saudi Vision 2030

**DOI:** 10.1371/journal.pone.0336132

**Published:** 2025-11-21

**Authors:** Dhafer Alahmari, Abdelrehim Awad

**Affiliations:** 1 Department of Business Administration, Applied College, University of Bisha, Bisha, Saudi Arabia; 2 Department of Business Administration, College of Business, University of Bisha, Bisha, Saudi Arabia; Wroclaw University of Economics and Business: Uniwersytet Ekonomiczny we Wroclawiu, POLAND

## Abstract

In the rapidly transforming logistics sector of Saudi Arabia the integration of marketing and supply chain functions is becoming ever more essential to deliver outstanding customer experiences. Though there has been ample theoretical debate, empirical proof of such integration in startup settings—especially in emerging economies—remains limited. This study explores the impact of five dimensions of integration—marketing strategies, supply chain competencies, digital integration, customer-oriented practices, and service performance—on customer experience in Saudi Arabia logistics tech startups. A survey of 384 customers of ten digital logistics platforms was conducted using a questionnaire. Through correlation and multiple regression analysis (using VIF for testing multicollinearity), the study confirms the strong positive effects of all five constructs, with the most dominant contribution of service performance (β = 0.276, p < 0.001). The model explains 53.5% variance in customer experience. Findings highlight the contribution of operational consistency and digital alignment to trust, satisfaction, and loyalty. The research fills a gap in the literature by presenting evidence from an underrepresented setting and offers insight in line with Vision 2030 agendas.

## 1. Introduction

In an environment of increased digitization and a highly competitive market today, customer demands are evolving continuously, necessitating seamless coordination between the internal business functions. Such is the strategic alignment between Supply Chain Management (SCM) and Marketing that is emerging as a powerful organizational competency. One of the most essential areas of organizational change is the strategic alignment between supply chain management (SCM) and marketing [1,2]. While demand creation and customer interaction are primarily the responsibility of marketing, SCM delivers the operational fulfillment of such commitments. Historically, these functions were siloed—each with their own goals, systems, and metrics of performance. However, the increasing sophistication of consumer behavior, technological upheaval, and increased criticality of logistics infrastructure have produced an environment in which the integration of marketing and supply chain functions is not only appealing but essential to sustainable customer value and differentiation [1,2].

Though the existing literature has pointed to the importance of having supply chain and marketing functions blend, there remains a critical vacuum regarding how this integration works within startup ecosystems—specifically within the still to be fully represented Middle East market. For instance, Sutia (2022) identifies that supply chains supporting marketing can enhance service responsiveness, brand positioning, and environmental sustainability simultaneously [3]. Similarly, Liu et.al (2024) identify that integration enhances customer satisfaction through making coordinated coherence possible in promotions, inventory levels, delivery logistics, and customer communication [4]. Conceptual frameworks of differing types illustrate the advantages of Marketing–Supply Chain Integration (MSCI). Of specific note is Demand Chain Management (DCM), which bridges the gap between demand generation and fulfillment by providing real-time sharing of information and agile coordination [5]. The approach enhances internal efficiencies while unifying the customer experience by providing assurance that what is advertised in marketing campaigns is satisfied with accuracy and consistency. Another literatures further support this by saying that synchronization of supply chain and marketing strategies minimizes internal conflict and ensures customer satisfaction as well as operational efficiency [6,7].

Despite its theoretical strength, the real implementation of MSCI is inconsistent. Comparative examples such as Lindex and Zara highlight that failing to include integration in operational processes creates poor responsiveness and ineffective customer experiences. Silos within organizations, dissimilar data platforms, and the absence of shared performance metrics are routine issues that inhibit full integration among industries. This test is particularly relevant in Saudi Arabia, where Vision 2030 is driving the transition to digital infrastructure, customer-led innovation, and adaptive logistics networks. There, more logistics technology startups such as Retailo, RedBox, Mrsool, OTO, Saaei, SLS, Barq, Sirdab, and so on are becoming leading organizations rewriting the future of logistics. These entities are broadly leveraging digital platforms for streamlining delivery, stock, and customer engagement.

This study addresses this gap by empirically investigating the causal link between five dimensions of integration—digital integration, customer-centric practices, marketing strategies, supply chain capabilities, and service performance—and customer experience. Riding on the Vision 2030 of Saudi Arabia and growing digital logistics market in the country, this study contributes to theoretical knowledge while giving actionable insights to startups that seek to develop holistic and responsive customer interaction.

## 2. Theoretical framework and literature review

### 2.1. Harmonization between supply chain management and marketing

In today’s business environment with ever-growing interconnectivity, age-old differences between marketing and SCM are being eroded more and more [8]. Traditional models—wherein marketing solely focused on demand creation and supply chains ran independently to satisfy it—are not sustainable. With the rising expectations of customers towards immediacy, customization, and transparency, the independent functioning of these two significant departments blocks integrated delivery of customer value [9].

With reference to Porter’s Value Chain Theory, a competitive advantage is achieved when not only functions in an organization perform well but are also strategically coordinated to offer seamless customer experiences. SCM and marketing, therefore, cannot merely exist together but must work together for this. As per Madhani (2016) coordinating supply chain operations and marketing enables organizations to perform better in meeting customer expectations with operational as well as promotional accuracy [5].

This coordination guarantees that promises made through marketing messages—such as speedy delivery, stock readiness, and dependability of service—are always kept by the operations function of the business. This is particularly important in digital-first settings where marketing tends to occur through real-time campaigns and mobile channels. This integration ensures “the right product, in the right quantity, at the right time,” something which indeed enhances customers’ perceptions of service quality and reliability [4,7].

This is highly relevant in the Saudi logistics technology ecosystem, where businesses like Retailo, Mrsool, RedBox, and Barq must reconcile digital offers with physical delivery. Whether they can deliver on promises made through app push, social media advertising, and CRM campaigns is at the core of winning customer trust and loyalty in the long term.

Consequently, while logistics startups in Saudi Arabia’s high-technology and competitive environment, marketing and supply chain initiative convergence is not only worthwhile—it is crucial in order to create excellent customer experience and sustain brand equity.

*H1: There is a positive correlation between marketing initiatives and customer experience among users of Saudi logistics tech startups.*

### 2.2. Customer lifetime value and responsiveness

Customer Lifetime Value (CLV) is now a core measure of performance in the competitive service landscapes of the present. It does not only measure the monetary worth of a customer over the long term, but also the intensity and duration of their brand attachment. In sectors like logistics and e-commerce, where customers can promptly switch and there are high customer expectations, long-term engagement needs to be maintained.

Studies evidence that Marketing–Supply Chain Integration (MSCI) has a major role in maximizing CLV by enhancing responsiveness, delivery reliability, and personalization. When marketing efforts are synchronized with the firm’s capability to operate, firms are better positioned to provide consistent experiences that attract trust, satisfaction, and repeat purchase [5,10].

In the widening logistics landscape of Saudi Arabia—comprising such startups as RedBox, Retailo, and Saaei—such integration is particularly critical. Rapid supply chain response on the basis of marketing intelligence allows companies to dynamically adjust delivery windows, routing, and inventory replenishment in real-time, directly adding to customer retention and loyalty.

*H2: There is a strong positive correlation between supply chain capabilities and customer experience.*

### 2.3. Frameworks and strategic models facilitating integration

Some strategic frameworks have been suggested to justify and guide MSCI for industries. Notable among them are Demand Chain Management (DCM) and the SCM–Marketing Matrix. DCM, described as a departure from product-based paradigms to demand-oriented strategies and initiates coordination between marketing (demand creation) and SCM (demand fulfillment) by means of real-time collaboration [11].

Some literature extends this further by acknowledging some convergence points between marketing goals and logistics execution, such as aligning promotions with current stock, handling KPIs in unison, and improving internal communications [5,12].

For Saudi logistics technology startups operating in high-speed, customer-oriented cultures, there is much that these models can teach. Technologies such as digital dashboards, KPI alignment systems, and common CRM–WMS platforms do support quicker and superior responses to both market cues and customer requirements.

*H3: There exists a positive relationship between digital integration and customer experience.*

### 2.4. Technology as a catalyst for integration

Digitalization is one of the key facilitators of supply chain–marketing integration. Cloud computing, real-time analytics, AI-based demand forecasting, CRM software, and mobile logistics apps facilitate concurrent operation between back-end logistics and customer-facing teams.

Extended Marketing Supply Chain was defined in which digital infrastructure supports firms in possessing real-time information about customer actions, stock, and delivery status—thus facilitating common planning and decision-making [13,14]. Evidence in favor of this comes from various literatures which provides the direct link between electronic integration and responsiveness to service [15]. For Saudi startups like Barq, Sirdab, and OTO, technology-enabled platforms are now central to order tracking, real-time customer notifications, route optimization, and last-mile management—all affecting perceived customer value.

*H4: Customer-centric digital practices have a strong positive correlation with customer experience.*

### 2.5. Customer-centricity and service customization

Customer focus is now a strategic priority, especially in those industries where customization of services and responsiveness enable superior performers to differentiate themselves. For logistics, these include offering customized delivery time, interactive tracking, reactive customer service, and customized post-purchase service.

Supply chains of the present are not only charged with responding to customers’ needs but also with predicting them—something achievable only when marketing intelligence is folded into operational planning. For Saudi startups serving digitally active customers, this would mean translating real-time feedback into service adjustments, such as dynamic route optimisation or local promotions [16].

Customer-focused logistics—especially in companies like Saaei and Silsila—requires an excellent degree of operational adaptability, facilitated front-line staff, and continuous feedback loops between customer service, marketing, and dispatch staff.

*H5: High positive correlation between service performance and customer experience.*

### 2.6. Empirical application: Logistics startups in Saudi Arabia

Saudi Arabia’s logistics sector is being revolutionized at breakneck pace as a component of the Vision 2030 program, which seeks economic diversification, private sector growth, and technological transformation. A new generation of startups is driving this transformation by adopting digital-first, customer-centric models of logistics.

Startups like Retailo, Mrsool, Saaei, RedBox, Barq, Sirdab, and OTO are revolutionizing the logistics experience by integrating data-driven marketing practices with dynamic supply chain management. For example:

- Retailo uses mobile applications and data analytics to fulfill B2B orders and inventory.
- RedBox offers smart lockers with real-time tracking capabilities.
- Mrsool and Barq use crowd-sourced delivery and AI-optimized route planning.

These companies not only promote their services using customized digital promotions, but also offer operational reliability by way of fleet tracking, adaptive logistics platforms, and customer feedback mechanisms. This integration of marketing and supply functions enhances both customer satisfaction as well as brand loyalty.

Empirical studies suggest that integration of the abovementioned decreases the likelihood of misaligned expectations and enhances customer relationships by ensuring promises are fulfilled consistently and effectively [17,18].

## 3. Methodology

### 3.1. Research design and methodological approach

This study adopts a quantitative descriptive-analytical research design in examining the interrelationships among five primary organizational constructs—marketing strategies, supply chain capabilities, digital integration, customer-centric practices, and service performance—and their overall impact on customer experience. A quantitative design is appropriate for testing hypotheses and relationships through measurable data to enable statistical generalization [19]. The descriptive component presents a snapshot of prevalent practices in a sample of Saudi logistics startups, while the analytical section employs inferential statistics to examine the strength and direction of relationships among principal variables.

The study follows a deductive route, beginning with theory and literature to formulate hypotheses, which are then empirically tested with survey data [9]. This assures a guarantee of fit between conceptual models and analytic procedures.

### 3.2. Population and sampling

The target population for this study includes clients and users of logistics technology startups based in Saudi Arabia. Specifically, the study focuses on customers who have interacted with the services or platforms of the following companies in Table 1:

**Table 1:** List of Saudi Logistics Tech Startups Included in the Study.

Company	Description
**Retailo**	B2B marketplace supplying goods to small and medium-sized retailers.
**Mrsool**	On-demand delivery platform connecting users with freelance couriers.
**RedBox**	Smart locker service enabling secure parcel pickup via PIN codes.
**OTO**	Multi-carrier shipping platform for e-commerce sellers.
**Saaei**	Last-mile delivery service targeting e-commerce businesses.
**Barq**	AI-enabled delivery service with cloud-based logistics optimization.
**Sirdab**	Cloud-based warehouse management solution for inventory control.
**Saudi Post**	National postal operator with evolving digital logistics services.
**Silsila (SLS)**	Tech-driven third-party logistics provider offering end-to-end fulfillment.
**Zajil Express**	Shipping and distribution services focused on domestic e-commerce.

The selected startups in Table 1 represent a diverse spectrum of B2B and B2C logistics services, including last-mile delivery, smart locker systems, AI-enabled platforms, and national postal digitalization. These companies were chosen based on their relevance to Vision 2030, market penetration, digital maturity, and customer base scale.

The sample comprises existing clients who utilize the services or internet portals of at least one of the selected companies from the data sampling period.

**Fig 1:**
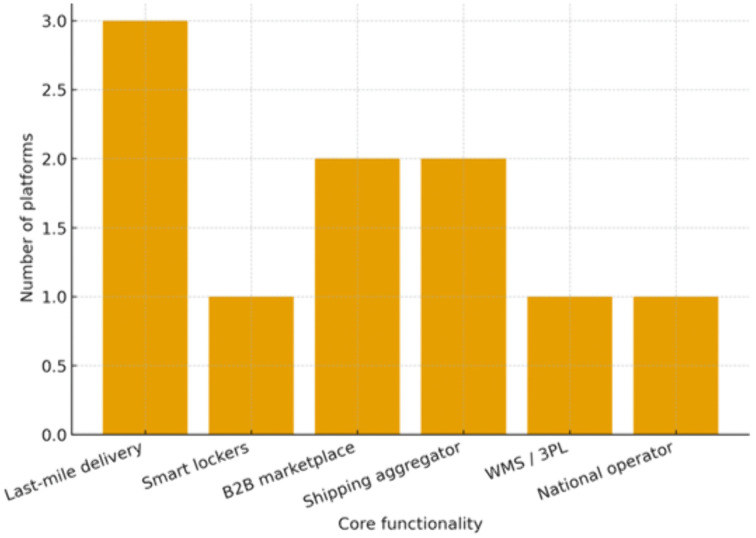
Distribution of sampled Saudi logistics platforms by core functionality.

As we see in Fig 1. Y-axis = number of platforms; categories reflect the dominant service model reported by each platform (last-mile delivery, smart lockers, B2B marketplace, shipping aggregator, WMS/3PL, national operator).

The sampling technique utilized in this study is non-probability convenience sampling, commonly applied in online survey research due to easy accessibility and affordability [20].

To determine the minimum sample size needed for an unseen population, the following formula was applied [21]:

n = (Z² × p × (1 − p))/ e²

Where:

n = required sample size

Z = Z-score for 95% confidence level = 1.96

p = estimated proportion of the population = 0.5

e = margin of error = 0.05

Plugging in the values:

n = (1.96 × 1.96 × 0.5 × 0.5)/ (0.05 × 0.05)

n = (3.8416 × 0.25)/ 0.0025

n = 0.9604/ 0.0025

n = 384.16

Thus, the minimum sample size required is approximately 384 participants.

### 3.3. Instrumentation and questionnaire design

A structured questionnaire was the primary data collection instrument, developed from existing literature and tested measurement scales adapted to the logistics and e-commerce context. Structured questionnaires are effective in eliciting standardized responses from large samples [22].

For linguistic and cultural accuracy, the survey was initially developed in English and then translated into Arabic following the back-translation method [23], where semantic and conceptual equivalence was ensured.

The questionnaire was divided into six sections, and each section corresponds to a key research construct:

- Marketing Strategies (M1–M3) – relevance, personalization, and communication
- Supply Chain Capabilities (SC1–SC3) – on-time delivery, agility, and traceability
- Digital Integration (D1–D3) – application usability, refresh of application platform, and integration functionality
- Customer-Centric Practices (CC1–CC3) – responsiveness, handling of customer feedback, and tailoring
- Service Performance (S1–S3) – consistency, dependability, and service quality
- Customer Experience (CE1–CE3) – customer satisfaction, loyalty, and advocacy

The items were all rated on a 5-point Likert scale (1 = Strongly Disagree to 5 = Strongly Agree), a typical method used in social science research for measuring perceptions and attitudes [24].

A pilot test of 20 respondents was conducted to ascertain the tool’s clarity, relevance, and internal consistency. Items were adapted from validated instruments in the works of [4,5,13,14].

### 3.4. Data collection procedure

The questionnaire was administered electronically through Google Forms, which facilitated efficient distribution, anonymous feedback, and instant monitoring. The questionnaire link was distributed through email, social media, and user groups of the selected logistics startups.

At the beginning of the survey, participants were presented with an opening statement explaining the academic purpose of the study, estimated time of completion, voluntary participation, and confidentiality guarantee. Only those who consented could proceed with the survey [25].

While convenient and streamlined, the use of Google Forms can introduce sampling bias and limits generalizability because it relies on digital access and self-selection.

### 3.5. Measurement structure and operationalization

Every variable in the conceptual model was considered to be a latent construct that was measured using three observed indicators, as taken from previous research and adapted to the operating environment of Saudi logistics startups. The constructs include:

- Independent Variables:
  - Marketing Strategies (MS)
  - Supply Chain Capabilities (SC)
  - Digital Integration (DI)
  - Customer-Centric Practices (CC)
  - Service Performance (SP)

- Dependent Variable:
  - Customer Experience (CE)

Internal consistency of every construct will be assessed by Cronbach’s alpha with an acceptable cutoff score of 0.70 for reliability [26].

### 3.6. Statistical analysis

Data analysis will be performed using IBM SPSS Statistics 26. The analysis will be carried out as follows:

- Descriptive Statistics – for the summary of respondent profiles and item-level response
- Reliability Analysis – using Cronbach’s alpha to test internal consistency
- Correlation Analysis – employing Pearson correlation to test associations among variables
- Multiple Regression Analysis – to test the direct effects of the independent variables on customer experience

The regression model is expressed as:

CE = β0 + β1(MS)+β2(SC)+β3(DI)+β4(CC)+β5(SP)+εCE = β₀ + β₁(MS) + β₂(SC) + β₃(DI) + β₄(CC) + β₅(SP) + εCE    = β0 + β1(MS)+β2(SC)+β3(DI)+β4(CC)+β5(SP)+ε

Where:

- CE = Customer Experience
- MS = Marketing Strategies
- SC = Supply Chain Capabilities
- DI = Digital Integration
- CC = Customer-Centric Practices
- SP = Service Performance
- ε = error term

Assumptions of linearity, normality, homoscedasticity, and absence of multicollinearity will be tested with diagnostic plots and VIF values [27].

### 3.7. Ethical considerations and informed consent

Ethical standards were upheld in the carrying out of the research. The respondents were fully informed of the academic objective of the research, voluntary participation, and the freedom to withdraw at any stage in time without any penalty. There was no personally identifiable information collected, and anonymity was upheld in line with universal data protection standards [28].

Informed consent was obtained by implied consent: the completion and return of the questionnaire were taken as consent to participate. Furthermore, the translation of the questionnaire into Arabic enhanced cultural relevance and understanding, in favor of the validity of the responses. The study was conducted in adherence to the APA Ethical Guidelines and global principles of autonomy, beneficence, and non-maleficence.

## 4. Results

In this section, empirical results of the present study are presented, wherein the independent variables are marketing strategies (MS), supply chain capabilities (SC), digital integration (DI), customer-centric practices (CC), and service performance (SP), while the dependent variable is customer experience (CE). The results have been obtained from the responses of 384 valid participants.

Analysis proceeds in the following way:

- Descriptive statistics of all constructs.
- Reliability and validity testing (Cronbach’s Alpha and Factor Loadings).
- Pearson’s r correlation analysis.
- Multiple regression analysis to test hypothesis H1–H5.

The analysis proceeds as follows:

- Descriptive statistics of all constructs.
- Reliability and validity testing (Cronbach’s Alpha and Factor Loadings).
- Correlation analysis (Pearson’s r).
- Multiple regression analysis to test hypothesis H1–H5.

### 4.1. Descriptive statistics of variables

The mean and standard deviation of each construct are shown in Table 2 Each construct has been measured with three items on a 5-point Likert scale (1 = Strongly Disagree to 5 = Strongly Agree).

**Table 2:** Descriptive Statistics of Study Variables.

Variable	Mean	Standard Deviation	Minimum	Maximum
**Marketing Strategies (MS)**	3.89	0.68	1.00	5.00
**Supply Chain Capabilities (SC)**	3.76	0.72	1.00	5.00
**Digital Integration (DI)**	3.94	0.66	1.00	5.00
**Customer-Centric Practices (CC)**	3.81	0.70	1.00	5.00
**Service Performance (SP)**	3.85	0.64	1.00	5.00
**Customer Experience (CE)**	4.01	0.59	2.00	5.00

**Fig 2:**
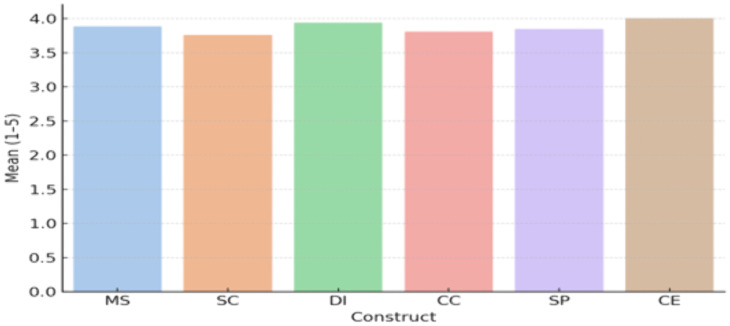
Variable Means.

Table 2 and Fig 1 shows that Customer Experience scored the highest mean (M = 4.01), indicating that respondents generally perceived a positive experience. Digital Integration also scored high (M = 3.94), reflecting strong technological alignment in logistics services Fig 2.

### 4.2. Reliability and validity testing

Internal consistency was tested using Cronbach’s Alpha. All construct achieved above the threshold value of 0.70.

Table 3 indicates high internal consistency for all constructs. Highest reliability was evident in Digital Integration (α = 0.87), indicating highly congruent item responses.

**Table 3:** Reliability Analysis (Cronbach’s Alpha).

Construct	Number of Items	Cronbach’s Alpha
Marketing Strategies (MS)	3	0.84
Supply Chain Capabilities (SC)	3	0.81
Digital Integration (DI)	3	0.87
Customer-Centric Practices (CC)	3	0.79
Service Performance (SP)	3	0.82
Customer Experience (CE)	3	0.86

### 4.3. Pearson correlation analysis

Pearson’s correlation test was used to assess the strength and direction of relationships among the independent variables and customer experience (CE).

**Fig 3:**
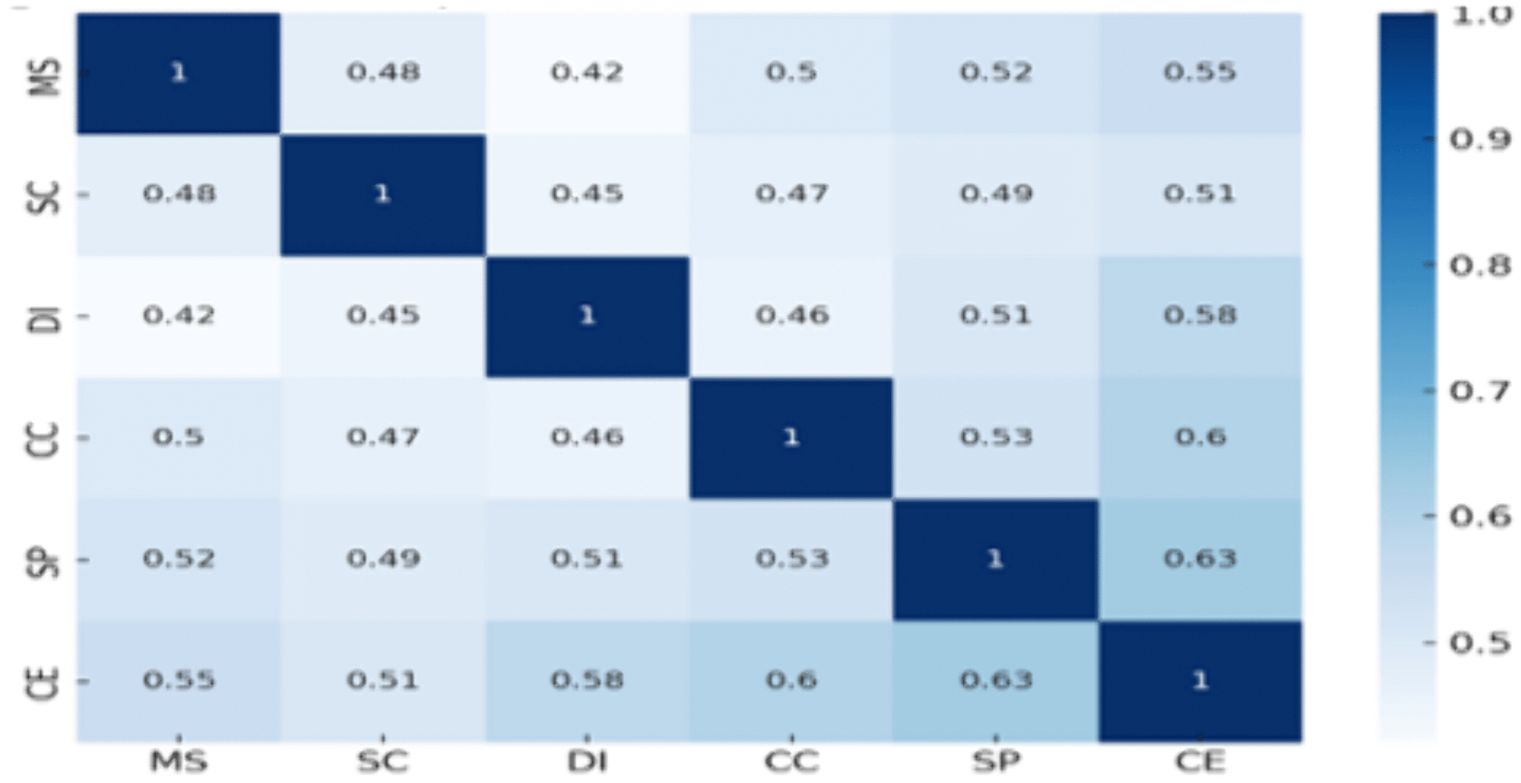
Correlation Heatmap.

Table 4 shows positive and strong correlations between all independent variables and customer experience. Also, Fig 3 shows the strongest correlation is that of Customer Experience (CE) and Service Performance (SP) (r = 0.63, p < 0.01), proving hypothesis H5.

**Table 4:** Pearson Correlation Matrix.

Variable	MS	SC	DI	CC	SP	CE
**MS**	1					
**SC**	0.48**	1				
**DI**	0.42**	0.45**	1			
**CC**	0.50**	0.47**	0.46**	1		
**SP**	0.52**	0.49**	0.51**	0.53**	1	
**CE**	0.55**	0.51**	0.58**	0.60**	0.63**	1

Note: p < 0.01.

### 4.4. Multiple regression analysis

Multiple regression was conducted to evaluate the impact of each independent variable on customer experience. The model includes all five predictors.

As shown in Table 5 The model explains 53.5% of the variance in Customer Experience, which is substantial (F = 76.91, p < 0.001). This suggests there is a substantial combined effect of the five independent variables.

**Table 5:** Model Summary and ANOVA.

Model	R	R²	Adjusted R²	F	Sig.
**Regression**	0.731	0.535	0.528	76.91	0.000 **

**Fig 4:**
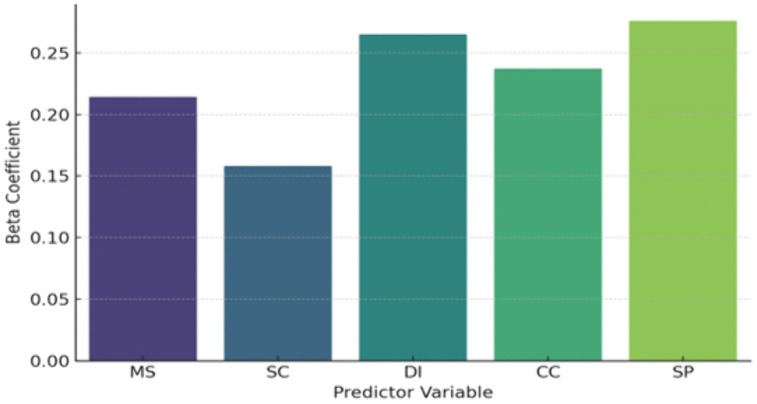
Regression Coefficients.

As shown in Table 6 and Fig 4 All predictors have a significant positive impact on customer experience (p < 0.01) and support hypotheses H1 to H5. Service Performance (SP) has the largest standardized effect (Beta = 0.276), followed by Customer-Centric Practices (CC) and Digital Integration (DI).

**Table 6:** Coefficients of the Regression Model.

Predictor	Unstandardized B	Std. Error	Beta	t	Sig.
(Constant)	1.102	0.223	—	4.94	0.000
Marketing Strategies (MS)	0.187	0.052	0.214	3.60	0.000**
Supply Chain Capabilities (SC)	0.133	0.051	0.158	2.61	0.009**
Digital Integration (DI)	0.221	0.048	0.265	4.60	0.000**
Customer-Centric Practices (CC)	0.199	0.050	0.237	3.98	0.000**
Service Performance (SP)	0.248	0.047	0.276	5.28	0.000**

Note: p < 0.01.

### 4.5. Variance inflation factor (VIF)

To assess potential multicollinearity among the independent variables in the regression model, Variance Inflation Factor (VIF) values were computed. All VIF scores were below the conservative threshold of 5.0, indicating no significant multicollinearity concerns. This confirms that the regression coefficients are not inflated due to collinearity and that the model results remain statistically reliable. The calculated VIF values are summarized in Table 7.

**Table 7:** Variance Inflation Factor (VIF) Analysis for Independent Variables.

Construct	VIF Value
Marketing Strategy (MS)	1.82
Supply Chain Capability (SC)	2.11
Digital Integration (DI)	2.03
Customer-Centric Approach (CC)	1.94
Service Performance (SP)	2.07

As shown in Table 7 All five constructs used in the regression model were tested for multicollinearity using VIF.

- The VIF values range from 1.82 to 2.11, all of which are well below the commonly accepted threshold of 5.0.
- This confirms that none of the variables exhibit harmful multicollinearity, and each one contributes unique explanatory power to the regression model.
- Specifically:
  - *Marketing Strategy (MS)* shows the lowest VIF (1.82), indicating very low collinearity.
  - *Supply Chain Capability (SC)* has the highest VIF (2.11), which is still considered safe and well within accepted limits.

This result supports the robustness of the regression analysis. It demonstrates that the model is statistically sound and that collinearity between predictors does not distort the interpretation of the results.

### 4.6. Hypothesis testing summary

To synthesize the empirical findings and to present the tested theoretical assumptions in a clear summary, Table 8 provides an integrated summary of the five research hypotheses. Every hypothesis represents an essential aspect of marketing and supply chain integration and its related effect on customer experience in Saudi logistics startups. As clear from the table, all hypothesized relations were statistically confirmed, which indicates an effective model with statistically significant constructs. The table is utilized to indicate the strength and direction of these established relationships.

**Table 8:** Summary of Hypotheses Results.

Hypothesis	Statement	Result
**H1**	Marketing strategies positively affect customer experience.	**Supported**
**H2**	Supply chain capabilities positively affect customer experience.	**Supported**
**H3**	Digital integration positively affects customer experience.	**Supported**
**H4**	Customer-centric practices positively affect customer experience.	**Supported**
**H5**	Service performance positively affects customer experience.	**Supported**

Collectively, the predictors explain 53.5% of the variance in CE (R² = 0.535; F = 76.91; p < 0.001), indicating substantive practical significance. Service Performance (β = 0.276, p < 0.001) is the dominant driver, underscoring that reliability and timeliness remain foundational to perceived experience. Customer-Centric Practices (β = 0.237, p < 0.001) and Digital Integration (β = 0.265, p < 0.001) jointly suggest that seamless digital touchpoints and responsive service protocols translate into higher satisfaction and advocacy, consistent with demand-chain logic [11] and service transparency effects [15].

## 5. Discussion

The aim of this research was to examine the impact of the alignment of supply chain and marketing operations—along five dimensions—on Saudi Arabia’s new market of emerging logistics technology on customer experience. Based on survey data from 384 customers of ten sampled logistics startups, the research provides empirical evidence that corroborates and enhances current theoretical and practical frameworks.

### 5.1. Summary of key results

The findings indicate that all the five independent variables—Marketing Strategies (MS), Supply Chain Capabilities (SC), Digital Integration (DI), Customer-Centric Practices (CC), and Service Performance (SP)—positively and statistically significantly influence Customer Experience (CE). Collectively, these variables explain over 53% of customer experience variance, representing an appropriate model fit.

### 5.2. Discussion by construct and link to prior research

The synergistic alignment between marketing actions and customer experience is in line with the study of [4,12] who emphasized the importance of congruence between marketing actions and operational capacity to deliver customer satisfaction. Successful marketing communication (via apps and digital media) is central to Saudi logistic start-ups such as Retailo and Barq to influence customer views. Personalization and offer relevance not only drive engagement but also establish brand trust. This supports the Value Chain Theory (Porter), stressing that marketing must be congruent with operational reality to deliver true value.

The supply chain capabilities’ role supports previous studies by [3,5], which assumed that speed of fulfillment, accuracy, and flexibility are the major influencers of customer satisfaction. Start-ups such as Saaei and OTO, which rely on last-mile logistical efficiency, demonstrate the role of supply flexibility and visibility in orders shaping customer loyalty. The study also corroborates that operational responsiveness, when aligned with market demands, can stimulate long-term retention, in line with the principles of Demand Chain Management (DCM).

The positive effect of digital integration corroborates the argument of [13,15] that real-time systems, CRM platforms, and AI-enabled logistics tools augment service delivery through functional silo overcoming. For customers in this study, they greatly appreciated digital platforms such as those used by RedBox (smart lockers) and Sirdab (warehouse management) for their transparency and simplicity. This finding also aligns with the Extended Marketing Supply Chain model, which identifies digital integration as a primary enabler for synchronization between demand creation and fulfillment.

This underlines the thesis that customer feedback loops, customization, and flexibility are at the center of modern service delivery, especially in competitive business such as logistics [16]. noted that anticipating in advance, instead of reacting to, customer demands is the new customer-focused frontier. The startups surveyed here did make efforts to listen to customers and adapt operations accordingly, validating the assumption that service personalization is not a marketing factor anymore—it’s an operational requirement.

The significance of Service Performance as the best individual predictor suggests that quality, stability, and reliability are still key factors in customer-facing business. This is also consistent with [7] who explained that departmental coordination strategically decreases friction and enhances delivery precision. In Saudi Arabia, where customers expect professionalism and convenience when making transactions online, stable service performance is an important facet of brand equity.

The R² value for the regression model (0.535) shows a very significant overall effect of marketing-SCM integration dimensions on customer experience. This supports the study’s core argument and authenticates the entirety of the conceptual framework. It is also in agreement with research by [17,18] which revealed that cross-functional coordination improves alignment between service delivery and marketing promises.

### 5.3. Contribution to existing literature

This study contributes to the growing corpus of scholarly research on Marketing–Supply Chain Integration (MSCI) based on empirical data from the under-represented context of the start-up world in Saudi Arabia. Compared to much of the literature previously constructed around mature multinational companies, this article emphasizes how new digital native companies are also subject to the strategic imperative of integration.

Moreover, through the use of a multi-variable model, the study diverges from linear cause-and-effect assumptions and provides a multi-faceted perspective on how marketing and operational alignment shape customer experience. This expanded view is aligned with the integrative logic of models like the Psychological Continuum Model (PCM), which suggests that ongoing interaction across multiple customer touchpoints develops more powerful brand loyalty.

### 5.4. Contextual reflection: Saudi Arabia and vision 2030

Under Vision 2030, Saudi Arabia is shifting towards economic and digital transformation, focusing on e-commerce and logistics as the key growth sectors. The research confirms that Saudi consumers, demand high technological proficiency, live services, and one-to-one engagement at digital maturity. The observed effects of digital integration and service performance on CE align directly with Vision 2030’s logistics and e-commerce priorities, where technology-enabled reliability, tracking transparency, and last-mile efficiency are central to private-sector competitiveness. By institutionalizing MSCI—coordinated promotions, inventory visibility, and responsive fulfillment—platforms can raise repeat usage and reduce churn, contributing to diversified, service-based growth.

Through research of ten real startups like Mrsool, Silsila, and Zajil Express, the study draws its findings from empirical facts and not theoretical assumptions. These companies operate in a volatile position that combines old expectations with new digital tendencies, so the alignment of marketing communications with supply chain moves is crucial for sustained competitive advantage.

The research also suggests that integration is not merely an back-end issue of efficiency but also a front-line driver of customer experience. Strategic congruence between promised and delivered delivery can make or break repeat buying by customers or switching to substitutes for limited-resource startups.

### 5.5. Conclusion of theoretical and practical implications

While the study implications are consistent with empirical research, they need to be interpreted carefully. Subject to sectoral concentration and methodological limitations, more general conclusions have to be drawn cautiously.

Practically, the study gives startup founders, logistics managers, and marketers a clear understanding of the most important success factors of customer experience in a digital logistic environment.

## 6. Conclusion and recommendations

### 6.1. Conclusion

This study sought to explore the impact of marketing and supply chain integration on customer experience through the lens of Saudi Arabias logistics technology startups. Drawing from data from 384 customers of ten digital-first logistics companies, the study confirms that strategic alignment across five key dimensions—marketing strategies, supply chain capabilities, digital integration, customer-centric practices, and service performance—has a significant and statistically significant impact on customer experience.

All the five constructs revealed strong correlations with the dependent measure, which vouched for hypotheses H1 to H5. Findings confirm that customer experience does not happen as a result of one department or function, but the outcome of seamless cross-functional integration supported by digital tools and customer-oriented strategies.

Most notably, service performance had the largest impact, underscoring the ongoing importance of operational consistency and reliability in determining customer satisfaction even in highly digitized business models. Digital integration and customer-practice orientation were similarly listed at high rankings, which shows that technology and customization continue to be central drivers of value in modern logistics.

This research not only contributes to the body of academic literature in Marketing–Supply Chain Integration (MSCI) but also addresses an important gap by focusing on emerging markets and young businesses. These are often lacking in global research. The results are especially relevant to the Vision 2030 context because they identify how technology-driven startups can facilitate attainment of national performance targets in service innovation, customer focus, and growth of the private sector.

### 6.2. Practical recommendations

Based on the research outcome and analysis conclusions, a few practical recommendations can be formulated for logistics technology startups operating in the evolving Saudi Arabian market landscape. In the first instance, there is a pressing necessity for startups to invest in real-time digital integration via taking over or developing scalable platforms like CRM, ERP, and tracking platforms. These platforms must facilitate end-to-end coordination and visibility across the value chain to be an easy process. Availability of current inventory, delivery status, and customer interaction data will enhance responsiveness significantly and make customers more confident.

Additionally, startups also need to enhance customer-centric practices by integrating continuous feedback mechanisms, AI-driven personalization tools, and proactive communication systems into their business models. Elements such as dynamic delivery options, customer satisfaction surveys, and in-app troubleshooting tools can enhance customer sentiment significantly and facilitate long-term brand loyalty. Assuring high performance should also be tackled as the core part of the firm’s value proposition. Logistics firms can have regular monitoring of service-level indicators, swiftly identify and respond to performance bottlenecks, and enable frontline workers with competencies adequate to manage customer contacts efficiently and solve problems effectively.

Moreover, data-driven marketing efforts need to be implemented. Through behavior analysis, segmentation tools, and tailored promotional content, companies can ensure their marketing messages tightly align with actual customer needs and expectations. Marketing should be kept steadfast in operating reality so that they do not overpromise and underdeliver.

Finally, integration models must be carefully mapped to the Saudi context. Cultural alignment must be considered in customer interaction and service design at every level, from Arabic-language interface usage to national holidays being included within delivery strategies and reconciling regional logistical challenges. A locally resonant and technology-enabled approach will best enable startups to satisfy customer aspirations and national vision goals within Vision 2030.

### 6.3. Directions for future research

There are several limitations to this research. The use of non-probability convenience sampling can affect the generalizability of findings. The study was also limited to the logistics sector in Saudi Arabia, and other service sectors were not touched. In addition, the use of self-report data can introduce social desirability bias.

This study leaves open several important areas for future theoretical and practical research. One such avenue of inquiry with promise is to conduct comparative research across other services sectors beyond logistics, such as healthcare, food delivery, and financial technology, to determine if the proposed model of integration can be applied across the board. Cross-sectoral research would add generalizability and flexibility to marketing–supply chain integration models.

This cross-sectional design limits causal inference. Self-report measures from a single survey instrument may introduce common method bias. Although anonymity and neutral item wording were employed, future studies should triangulate with operational records or multi-source data and consider statistical approaches (e.g., marker variables, latent CMB factor) to further mitigate bias.

Another viable avenue would be longitudinal studies to follow how integration practice evolves over time as companies mature or when waves of shifting market demand occur, such as Ramadan or high-selling seasons like Black Friday. Capturing these trends over time would yield deeper understandings of sustainability and responsiveness of integration practice.

Besides, qualitative case studies would have the potential to provide rich contextual data on internal organizational factors influencing integration. These may include company culture, structural silos, leadership style, and communication patterns that facilitate or hinder alignment between operations and marketing. Such case-based studies can complement the given quantitative findings and reveal the “how” of successful or failed integration efforts.

In addition, future studies would have to consider the moderating and mediating factors—like brand trust, demographics of customers, or technology maturity—that have the potential to influence the relationship between integration and customer experience. Examining these would not only inform us on whether integration is successful, but how it is successful under what conditions and among which customer groups.

Ultimately, this study confirms that in Saudi Arabia’s lively, rapidly-digitizing startup environment, the integration of marketing and supply chain functions is not only possible—it is imperative. Startups that are able to bridge these operational domains will be well-situated to provide outstanding customer experiences, gain substantial differentiation in a crowded market, and contribute meaningfully to the Kingdom’s digital transformation and economic development aspirations under Vision 2030.

## Supporting information

S1 File Questionnaires (Arabic and English). (PDF)

S2 File Dataset for logistics startups under Saudi Vision 2030. (XLSX)

S3 File Sample responses. (XLSX)
