# Supplementary material for: Strategic integration of marketing and supply chain functions for superior customer experience: Insights from logistics startups under Saudi Vision 2030
Source: PLoS One. 2025 Nov 21;20(11):e0336132. doi: 10.1371/journal.pone.0336132 (PMC12637983; doi:10.1371/journal.pone.0336132)
Supplement: S1 File — (PDF) [file pone.0336132.s001.pdf]

مقدمة:

عزيزي المشارك/عزيزتي المشاركة،
هذا الاستبيان جزء من دراسة أكاديمية تهدف إلى قياس العلاقة بين استراتيجيات التسويق، قدرات سلسلة الإمداد، التكامل الرقمي، وتجربة العميل في شركة "طرود". نرجو تعبئة الاستبيان بكل صدق، علمًا أن إجاباتكم ستُعامل بسرية تامة ولن تُستخدم إلا لأغراض البحث العلمي.

مقياس التقدير:

**الرمز**	**التقدير**
1	لا أوافق بشدة
2	لا أوافق
3	محايد
4	أوافق
5	أوافق بشدة

أولًا: استراتيجيات التسويق

رقم	العبارة	5	4	3	2	1
M1	تقدم الشركة عروضًا ترويجية تناسب احتياجاتي.	☐	☐	☐	☐	☐
M2	تتواصل الشركة معي بطرق تناسب تفضيلاتي.	☐	☐	☐	☐	☐
M3	تقدم الشركة محتوى تسويقي مخصص لي.	☐	☐	☐	☐	☐

ثانيًا: قدرات سلسلة الإمداد

رقم	العبارة	5	4	3	2	1
SC1	تسلمني الشركة الطلبات في الوقت المحدد.	☐	☐	☐	☐	☐
SC2	توجد مرونة في تعديل الطلب أو العنوان بعد الشراء.	☐	☐	☐	☐	☐
SC3	توفر الشركة معلومات تتبع دقيقة ومحدثة.	☐	☐	☐	☐	☐

ثالثًا: التكامل الرقمي

رقم	العبارة	5	4	3	2	1
D1	أستطيع متابعة الطلب من خلال تطبيق أو موقع إلكتروني بسهولة.	☐	☐	☐	☐	☐
D2	تجربة استخدام المنصة الرقمية سلسة وسهلة.	☐	☐	☐	☐	☐
D3	يتم تحديث حالة الطلب بشكل لحظي.	☐	☐	☐	☐	☐

رابعًا: الممارسات المتمحورة حول العميل

رقم	العبارة	5	4	3	2	1
CC1	تُعاملني الشركة كعميل مميز.	☐	☐	☐	☐	☐
CC2	تتيح لي الشركة خيارات متعددة تناسب احتياجاتي.	☐	☐	☐	☐	☐
CC3	تتفاعل الشركة مع ملاحظاتي أو شكاواي بسرعة.	☐	☐	☐	☐	☐

خامسًا: أداء الخدمة

رقم	العبارة	5	4	3	2	1
S1	مستوى الخدمة المقدمة يلبي توقعاتي.	☐	☐	☐	☐	☐
S2	الخدمة متسقة في جميع المرات التي استخدمتها فيها.	☐	☐	☐	☐	☐
S3	يوجد توافق بين ما تَعِد به الشركة وما يتم تقديمه فعلًا.	☐	☐	☐	☐	☐

سادسًا: تجربة العميل

رقم	العبارة	5	4	3	2	1
CE1	أشعر بالرضا العام عن تجربتي مع الشركة.	☐	☐	☐	☐	☐
CE2	أوصي الآخرين باستخدام خدمات الشركة.	☐	☐	☐	☐	☐
CE3	أشعر بالولاء للشركة بسبب تجربتي الإيجابية.	☐	☐	☐	☐	☐

Introduction:

Dear participant,
This survey is part of an academic study aiming to measure the impact of marketing strategies, supply chain capabilities, and digital integration on customer experience at **Torod**. Your participation is voluntary, and your responses will be kept strictly confidential.

Likert Scale:

Value	Interpretation
1	Strongly Disagree
2	Disagree
3	Neutral
4	Agree
5	Strongly Agree

Section 1: Marketing Strategies

No.	Statement	1	2	3	4	5
M1	The company offers promotions that match my needs.	☐	☐	☐	☐	☐
M2	The company communicates with me through preferred channels.	☐	☐	☐	☐	☐
M3	The company provides personalized marketing content.	☐	☐	☐	☐	☐

Section 2: Supply Chain Capabilities

No.	Statement	1	2	3	4	5
SC1	The company delivers my orders on time.	☐	☐	☐	☐	☐
SC2	There is flexibility in modifying the order or address after purchase.	☐	☐	☐	☐	☐
SC3	The company provides accurate and updated tracking information.	☐	☐	☐	☐	☐

Section 3: Digital Integration

No.	Statement	1	2	3	4	5
D1	I can track my order easily through the app or website.	☐	☐	☐	☐	☐
D2	The digital platform is easy and smooth to use.	☐	☐	☐	☐	☐
D3	Order status updates are provided in real time.	☐	☐	☐	☐	☐

Section 4: Customer-Centric Practices

No.	Statement	1	2	3	4	5
CC1	The company treats me as a valued customer.	☐	☐	☐	☐	☐
CC2	The company offers various options tailored to my needs.	☐	☐	☐	☐	☐
CC3	The company responds promptly to my feedback or complaints.	☐	☐	☐	☐	☐

Section 5: Service Performance

No.	Statement	1	2	3	4	5
S1	The level of service meets my expectations.	☐	☐	☐	☐	☐
S2	The service is consistent every time I use it.	☐	☐	☐	☐	☐
S3	There is alignment between the company’s promise and actual delivery.	☐	☐	☐	☐	☐

Section 6: Customer Experience

No.	Statement	1	2	3	4	5
CE1	I feel generally satisfied with my experience with the company.	☐	☐	☐	☐	☐
CE2	I would recommend the company’s services to others.	☐	☐	☐	☐	☐
CE3	I feel loyal to the company due to the positive experience.	☐	☐	☐	☐	☐
